# Spectrally-Resolved Synergy in Photothermal Catalysis: A Temperature-regulated Transition of Hot-electron Transfer for Methanol Steam Reforming

**DOI:** 10.1021/acsomega.6c05080

**Published:** 2026-06-18

**Authors:** Lifeng Xu, Chenghao Yao, Rui Lang, Lei Li, Zhan Lin

**Affiliations:** † College of Chemical and Biological Engineering, Zhejiang University, Hangzhou 310058, China; ‡ School of Chemical Engineering and Light Industry, 47870 Guangdong University of Technology, Guangzhou 510006, China

## Abstract

Photothermal catalysis promises to convert solar energy into fuels with the assistance of thermal energy, yet the synergy between light and heat remains poorly understood. Here, this work deconstructs this synergy in methanol steam reforming over Pt/TiO_2_. The catalyst achieves an H_2_ production rate of 688 mmol g^–1^ h^–1^ under UV/visible coupled illumination, driven by a nonlinear Arrhenius behavior. Spectral decoupling reveals a distinct wavelength-dependent thermal response under different temperatures. The UV band gap excitation of TiO_2_, although accelerating the generation of formaldehyde, remains kinetically limited by its accumulation; in contrast, visible metal interband excitation on Pt induces hot-electron injection to generate sufficient driving force to accelerate formaldehyde dissociation (>170 °C). Strong excitation (405 nm) generates sufficient driving force, whereas weak excitation (450 nm) fails to fully overcome the energy threshold required to overcome this kinetic trap. These findings propose a thermodynamic regulation model that selectively amplifies hot-electron channels, establishing a strategy to rationally harness the solar spectrum.

## Graphical abstract

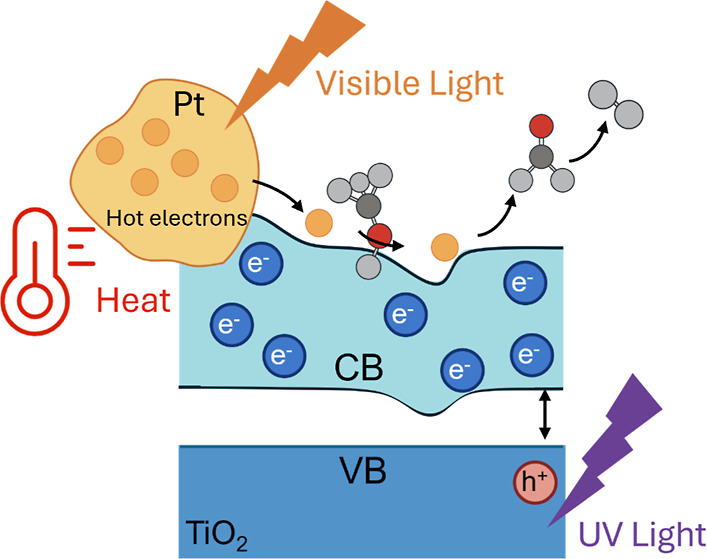

## 1 Introduction

The rising global demand for clean and sustainable energy has propelled the search for efficient hydrogen (H_2_) production technologies.^1^ Among various H_2_ production methods, methanol steam reforming (MSR, CH_3_OH + H_2_O → CO_2_ + 3H_2_) stands out as a particularly promising route due to its high hydrogen-to-carbon ratio, mild operating conditions, and the ease of storing and transporting liquid methanol.^2,3^ Conventional MSR with thermal catalytic process relies on significant thermal energy input to achieve high conversion rates.^4,5^

To mitigate the high energy consumption of traditional thermal catalysis, the field is evolving from simple photocatalysis toward photon–phonon codriven catalysis as an innovative strategy that utilizes light, particularly abundant solar energy, to drive chemical reactions.^6–9^ This approach offers the potential for lower operating temperatures and enhanced energy efficiency. TiO_2_, a benchmark semiconductor photocatalyst, is widely used but fundamentally limited by its band gap, allowing only the ultraviolet (UV) portion of the solar spectrum.^10^

To extend light harvesting into the visible range, a prominent strategy involves integrating Pt nanoparticles, which are dominated by strong interband transitions.^11^ Integrating Pt nanoparticles introduces multiple energy conversion pathways. Beyond acting as catalytic active sites, Pt nanoparticles can harvest visible light via strong interband transitions to generate energetic hot electrons—a nonthermal effect—or decay into localized heat. In a catalytic system over Pt/TiO_2_, while our previous study demonstrated an overall temperature-triggered mechanism switch, it treated the full-spectrum illumination as a single entity,^12^ the precise roles of decoupled photon energies and their specific interactions with reaction intermediates remain largely unknown. UV-driven semiconductor band gap excitation, visible-driven hot-electron generation, and externally supplied thermal energy all coexist; their interplay is complex. A critical and yet poorly understood question is how these distinct energy inputs interact, specifically whether they contribute additively or if a more complex, synergistic mechanism is at play. More importantly, it remains unclear whether this thermal activation effect is universally applicable to all photoexcited carriers or strictly governs specific energetic channels.^13^ Deciphering the specific roles and interplay of electronic effects and thermal energy is paramount for the rational design of next-generation, high-efficiency solar-driven catalysts.

Herein, this study moves beyond the phenomenological observation of photoenhancement to deconstruct the mechanistic interplay between photonic and thermal energy in MSR over a Pt/TiO_2_ catalyst. A distinct nonlinear Arrhenius behavior is uncovered, crucially demonstrating that this mechanism is intrinsically wavelength-dependent. By integrating precision-controlled spectral decoupling with in situ spectroscopy, the contributions of the semiconductor band gap excitation (UV range) are distinguished from the metal-derived hot-electron injection (visible range). This finding reveals that thermal energy does not simply provide general rate enhancement but specifically activates the high-flux hot-electron pathway at elevated temperatures. All these reveal that the synergy is driven by the temperature-enabled breaking of a specific formaldehyde-related kinetic restriction, a process inaccessible to room-temperature photocatalysis. This work provides a unified “dual-channel” framework that rationalizes the supra-additive behavior of light and heat, offering a blueprint for designing high-efficiency, full-spectrum solar fuel catalysts.

## 2 Experimental Section

### 2.1 Catalyst Synthesis

The TiO_2_ support was synthesized via a sol–gel-hydrothermal strategy. Tetrabutyl orthotitanate (10 mL) was initially dispersed in ethanol (20 mL) containing acetic acid (3 mL), followed by the addition of an aqueous ethanol mixture (10 mL ethanol and 3 mL water) and stirring for 30 min. The resulting suspension underwent hydrothermal treatment in a Teflon-lined stainless steel autoclave at 170 °C for 30 h, after which the product was isolated by centrifugation, dried for 12 h, and annealed at 400 °C (5 °C/min) for 2 h.

The Pt/TiO_2_ catalyst were prepared via a photodeposition strategy. Specifically, a calculated volume of aqueous K_2_PtCl_6_ (10 mg/L) was introduced into a methanolic suspension of TiO_2_ (2 mL). The resulting mixture was uniformly cast onto a quartz substrate, dried, and irradiated under an Xe lamp (300 mW cm^–2^, Aulight) for 30 min to induce Pt reduction. The final Pt loading was quantified as 0.0545 wt % by ICP–MS analysis (Table S1). The ultralow Pt loading of 0.05 wt % was chosen to maximize the atomic dispersion of the noble metal, prevent high-temperature sintering, and avoid the optical shielding effect that blocks UV excitation of the TiO_2_ support.

### 2.2 Catalytic Performance Evaluation

Catalytic performance was evaluated in a photothermal reactor (PLR-GPTR50T, PrefectLight, China), where a quartz sheet loaded with 30 mg of catalyst was suspended via a glass holder. A methanol feedstock (methanol/water = 3:1) was introduced into the reactor using N_2_ as the carrier gas at ambient pressure. Experiments covered photocatalytic (PC), thermal catalytic (TC), and photothermal catalytic (PTC) modes over a temperature range of 30–190 °C. The reaction mixture flowed through the reactor with a top quartz window, which allows the light to directly irradiate the horizontal catalyst surface. For the PC and PTC tests, the cell was irradiated by a 300 W Xe lamp (CEL-HXF300, Aulight, China), with the light intensity calibrated to 500 mW cm^–2^ using an optical power meter (CEL-NP2000). The tip of the thermocouple was positioned in direct contact with the bottom of the quartz substrate to accurately monitor the catalyst operating temperature. To ensure a constant photon flux across different irradiation wavelengths, the applied light power density (*P*) for each LED source was carefully adjusted to be inversely proportional to its specific wavelength (λ). This calibration is based on the physical relationship *P* = *Np* × *hc*/λ, where *Np* represents the target photon flux, *h* is Planck’s constant, and *c* is the speed of light. Accordingly, the power densities were set to 221, 200, and 180 mW cm^–2^ for the 365, 405, and 450 nm light sources, respectively. Hydrogen production was quantified via gas chromatography (GC) analysis of the headspace gas.

The hydrogen production rate (STY) calculation was performed using the following equation

1 $$STY=\frac{mmol\;of\;H_{2}}{mass\;of\;catalyst\times time}$$

The synergistic enhancement calculation was performed using the following equation

2 $$synergy\;\left(\%\right)=\frac{STY\text{\_}combined-\left(\sum STY\text{\_}individual\right)}{\sum STY\text{\_}individual}\times 100\%$$

### 2.3 Catalyst Characterization

The powder X-ray diffraction (XRD) patterns of the catalysts were collected on a Rigaku-MiniFlex 600 X-ray diffractometer using Cu Kα (λ = 1.54059 Å) radiation in the 2θ range from 20° to 90° at a scan rate of 1° per minute.

The Scanning electron microscope (SEM) of the samples was performed on a Hitachi SU8220 field-emission scanning electron microscope.

The Transmission electron microscopy (TEM) was measured on a JEOL-JEM 2200FS electron microscope (200 kV) to display the morphology of the catalysts, and EDX elemental maps were conducted. Before the test, the samples were ultrasonically dispersed in ethanol and then dropped on a carbon-coated copper grid.

A three-electrode cell was employed to measure the photocurrent and the cyclic voltammetry (CV) on an electrochemical analyzer (CHI-760E, China) electrochemical workstation. The working electrode was made according to the following procedure. After 10 mg catalyst distributed in the solution of 1 mL ethanol and 20 μL Nafion solution, the dispersed suspension is evenly smeared on 35 mm × 35 mm conductive glass. The conductive glass was dried at 100 °C for 4 h after drying in the air. Then the conductive glass was cooled to room temperature. At room temperature, photocurrent generation curves were gained by an electrochemical workstation with the 300 W xenon lamp as the light source, which was 5 cm from the working electrode. The light transmission area of the quartz electrolytic cell was 3.14 cm^2^, in which 0.1 M Na_2_SO_4_ solution was used as the electrolyte. The bias current set during the photocurrent test was 0 V, and intermittent visible light irradiation was performed every 30 s.

The gas chromatograph (GC) from methanol reforming was examined by a gas chromatograph (FULI instruments, FL9790) with a thermal conductivity detector (TCD) equipped with a Trace PLOT TG-BOND Sieve 5A GC column (length: 50 m; diameter: 0.53 mm; film thickness: 10 μm).

The in situ ultraviolet–visible spectroscopy (in situ UV–vis) was performed on SHIMADZU UV3600iplus. The catalyst sample was measured at room temperature and 190 °C in N_2_.

The in situ electron paramagnetic resonance (EPR) was performed on a Bruker EMXplus-10/12 under illumination at 365 and 450 nm light at room temperature.

The in situ X-ray photoelectron spectroscopy (XPS) characterizations were performed on a Thermo Fisher ESCALAB 250Xi equipped with an in situ reactor, using Al Kα radiation as the excitation source (*hv* = 1486.6 eV), 8 × 10^–10^ mbar analysis chamber vacuum level, 12.5 kV working voltage, and 16 mA filament current. The passing energy was 30 eV with a step of 0.1 eV, and the signal accumulation was approximately ten cycles. Before collecting data, the catalyst is stable at each stage for 30 min of illumination with 365 and 450 nm light. The binding energy was corrected using C 1s (284.8 eV) as a reference.

The in situ diffuse reflectance infrared Fourier transform spectroscopy (DRIFTs) spectra were measured using a PerkinElmer Spectrum 3 FT-IR spectrometer equipped with a mercury–cadmium-telluride (MCT) detector and Praying Mantis Diffuse Reflection Accessory (HARRICK, UK) equip with an in situ infrared reaction cell (In situ High-tech), the light was brought in by a 300 W xenon lamp and optical fiber combination (Aulight, China). 112 scans (∼1 min) with a resolution of 4 cm^–1^ were recorded in the range of 4000–1000 cm^–1^ for all spectra, if not specified. The sample cup was first filled with ∼2 mg of quartz sand (1–2 mm), on top of which 10 mg of powder samples were placed. All samples were in situ pretreated at 400 °C for 30 min under 50 mL/min N_2_ flow before any measurement. Methanol/water vapor mixture was introduced into the reaction cell by bubbling N_2_ carrier gas through a saturator containing CH_3_OH/H_2_O (3:1) solution. Before introducing the reaction gas mixture, the catalyst was heated to the target reaction temperatures (150 and 190 °C) under a continuous flow of N_2_ (30 mL/min). The system was held at the target temperature for 30 min to reach thermal equilibrium and remove surface impurities. Following the background acquisition, the methanol and water vapor mixture was introduced into the cell to initiate the in situ measurements.

## 3 Result and Discussion

### 3.1 Structural and Morphological Characterization of the Pt/TiO_2_ Catalyst

The Pt/TiO_2_ catalyst was prepared by the hydrothermal method, followed by loading ∼2.0 nm Pt(111) species through photodeposition. Microscopic inspection via TEM and HAADF-STEM (Figures 1a,b, and S1) reveals that the highly dispersed Pt nanoparticles are successfully loaded on the TiO_2_ surface. The bare TiO_2_ support consists of agglomerated nanoparticles, providing a robust porous network. Notably, the morphological features and the precise dimensions of this optimized Pt/TiO_2_ model catalyst are highly consistent with the comprehensive structural baseline established in our recent foundational study.^12^ The X-ray diffraction (XRD) patterns displayed in Figure 1c demonstrate that the characteristic diffraction peaks of all the samples correspond to the anatase TiO_2_ structure. The absence of the peaks related to Pt species confirms the low concentration of Pt and the highly uniform dispersion of Pt species. This exceptional structural and morphological robustness serves as a critical prerequisite, ensuring that the catalytic performance metrics reported herein accurately reflect the material’s intrinsic activity, unperturbed by structural degradation or reconstruction artifacts.

**Figure 1:**
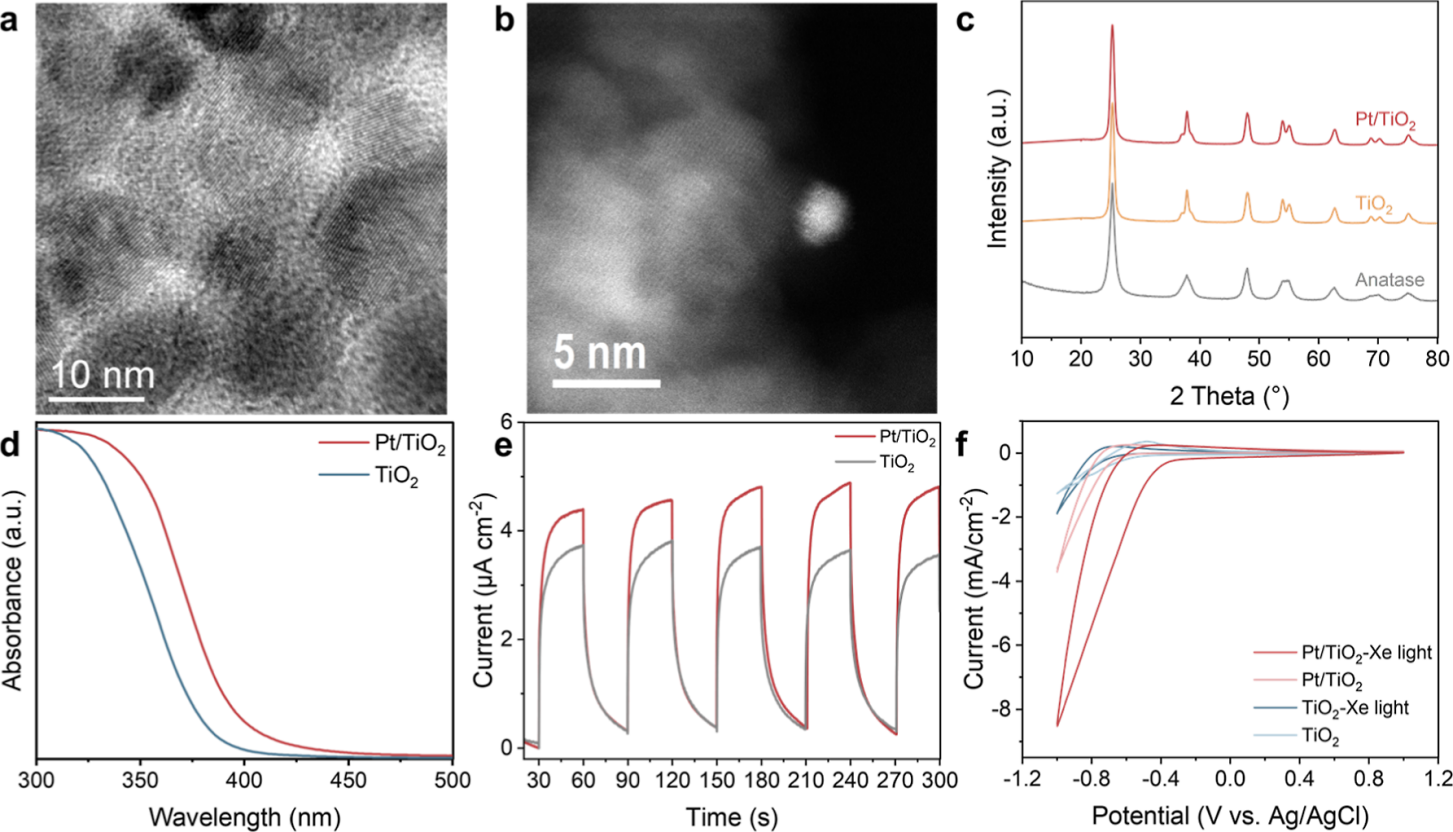
Characterization of the catalysts. (a,b) TEM and HAADF-STEM images of the Pt/TiO_2_. (c) XRD patterns of the catalysts. (d) UV–vis spectra of the catalysts. (e) Photocurrent response of the TiO_2_ and Pt/TiO_2_. (f) CV spectra of the Pt/TiO_2_.

To explore the optical properties of the catalyst, UV–vis measurements were employed to probe the optical absorption properties (Figure 1d). The spectrum reveals distinct absorption features across the UV–visible range, corresponding to the strong intrinsic band gap absorption of the TiO_2_ support. In the visible region, the Pt/TiO_2_ exhibits a broad absorption tail extending from the UV edge, which is attributed to the strong interband transitions of the ∼2.0 nm Pt nanoparticles.^11^ While broad light absorption is a prerequisite, the efficient separation of photogenerated carriers is equally vital. Therefore, photoelectrochemical measurements were employed to probe the charge carrier dynamics (Figure 1e). The Pt/TiO_2_ catalyst exhibits a markedly superior photocurrent response compared to the bare TiO_2_ support. This substantial enhancement provides compelling evidence that the dispersed Pt nanoparticles function as effective charge separation centers, which drastically suppress the recombination of photogenerated electron–hole pairs and promote their spatial separation.^14^

To further probe the thermodynamic driving force and charge transfer kinetics, cyclic voltammetry (CV) measurements were conducted (Figure 1f). In the dark, the Pt/TiO_2_ catalyst exhibits a moderate increase in cathodic current compared to pristine TiO_2_, attributed to the intrinsic electrocatalytic activity of metallic Pt for proton reduction.^15^ However, under Xe lamp irradiation, the Pt/TiO_2_ electrode displays a drastic enhancement in current density (up to ∼8 mA cm^–2^ at −1.0 V) and a distinct positive shift in the reduction onset potential. This behavior serves as unambiguous evidence that the Pt nanoparticles act as efficient charge separation and catalytic centers.^16,17^ They not only accumulate photogenerated electrons but also energetically facilitate the reduction half-reaction (H^+^ to H_2_) by lowering the kinetic overpotential.^18^ This electrochemical synergy perfectly mirrors the catalytic enhancement observed in the gas-phase MSR reaction.

To elucidate the structural origin of this robust redox capability and probe the metal–support interaction state, the reducibility of the catalyst was investigated by H_2_-TPR (Figure S2). While pure TiO_2_ shows no significant hydrogen consumption, the Pt/TiO_2_ catalyst exhibits a distinct reduction peak centered at ∼280 °C. Crucially, this reduction temperature is significantly higher than that of bulk Pt oxides, indicative of a strong metal–support Interaction (SMSI) that stabilizes the Pt species.^19,20^ Recent kinetic studies suggest that such TPR profiles are also intimately governed by the hydrogen spillover efficiency at the metal–support interface,^21^ implying that an intimate contact between Pt and TiO_2_ facilitates H atom transport. The high reduction temperature (∼280 °C) suggests that the catalyst cannot be fully activated by heat alone at the operating temperature (190 °C). Therefore, light irradiation is essential to bridge this energy gap. This underscores the necessity of the photothermal synergy, where photon energy compensates for the thermal deficit to drive the catalytic turnover. Complementing the reducibility studies, H_2_-TPD profiles were recorded to evaluate the hydrogen adsorption capability of the catalyst (Figure S3). The bare TiO_2_ support exhibits a minor, broad desorption feature centered around 108 °C, which is attributed to hydrogen species adsorbed on surface oxygen vacancies or defect sites induced during pretreatment.^22^ In contrast, the Pt/TiO_2_ catalyst displays a markedly enhanced and sharper peak that is shifted to a lower temperature (∼96 °C). This intensified signal is distinct from the background support and is attributed to the desorption of chemically adsorbed hydrogen atoms from the exposed metallic Pt surface at a lower temperature.^23^ The presence of this peak confirms that the Pt nanoparticles provide critical sites for the recombination of atomic hydrogen into molecular H_2_.^24^ Efficient removal of surface hydrogen (H) as H_2_ gas is thermodynamically critical for driving the continuous dehydrogenation equilibrium of methanol intermediates (e.g., methoxy to formate).

With the intrinsic physicochemical properties (structure, reducibility, and adsorption capacity) of the catalyst fully established, the accuracy of the photothermal kinetic measurements was subsequently validated by excluding potential thermal and optical artifacts. As shown in Figure S4, the introduction of illumination induces negligible temperature fluctuation (<1 °C) compared to the bulk reactor set points. The temperature profiles under illumination confirm that the external heating source dominates the thermal environment. These control experiments collectively substantiate that the supra-additive H_2_ production stems from intrinsic nonthermal synergistic mechanisms rather than physical heating or optical artifacts.

### 3.2 Catalytic Performance and Kinetic Analysis of Photothermal Synergy

The photothermal catalytic activity was evaluated using methanol steam reforming (MSR). At an operating temperature of 190 °C, the catalyst delivers a remarkable H_2_ production rate of 210 mmol g^–1^ h^–1^ under a full-spectrum Xe lamp (500 mW cm^–2^) (Figure 2a). To deconstruct the contributions of distinct spectral bands to this aggregate performance, a wavelength-dependent activity map was constructed using specific optical filters. The performance reflects the spectral response trend under filtered light intensities, rather than normalized photon flux conditions. As expected, irradiation with 365 nm UV light yields the highest monochromatic activity (35 mmol g^–1^ h^–1^), originating from the direct band gap excitation of the anatase TiO_2_ (*E*_g_ ≈ 3.2 eV) (Figure S5).^25^

**Figure 2:**
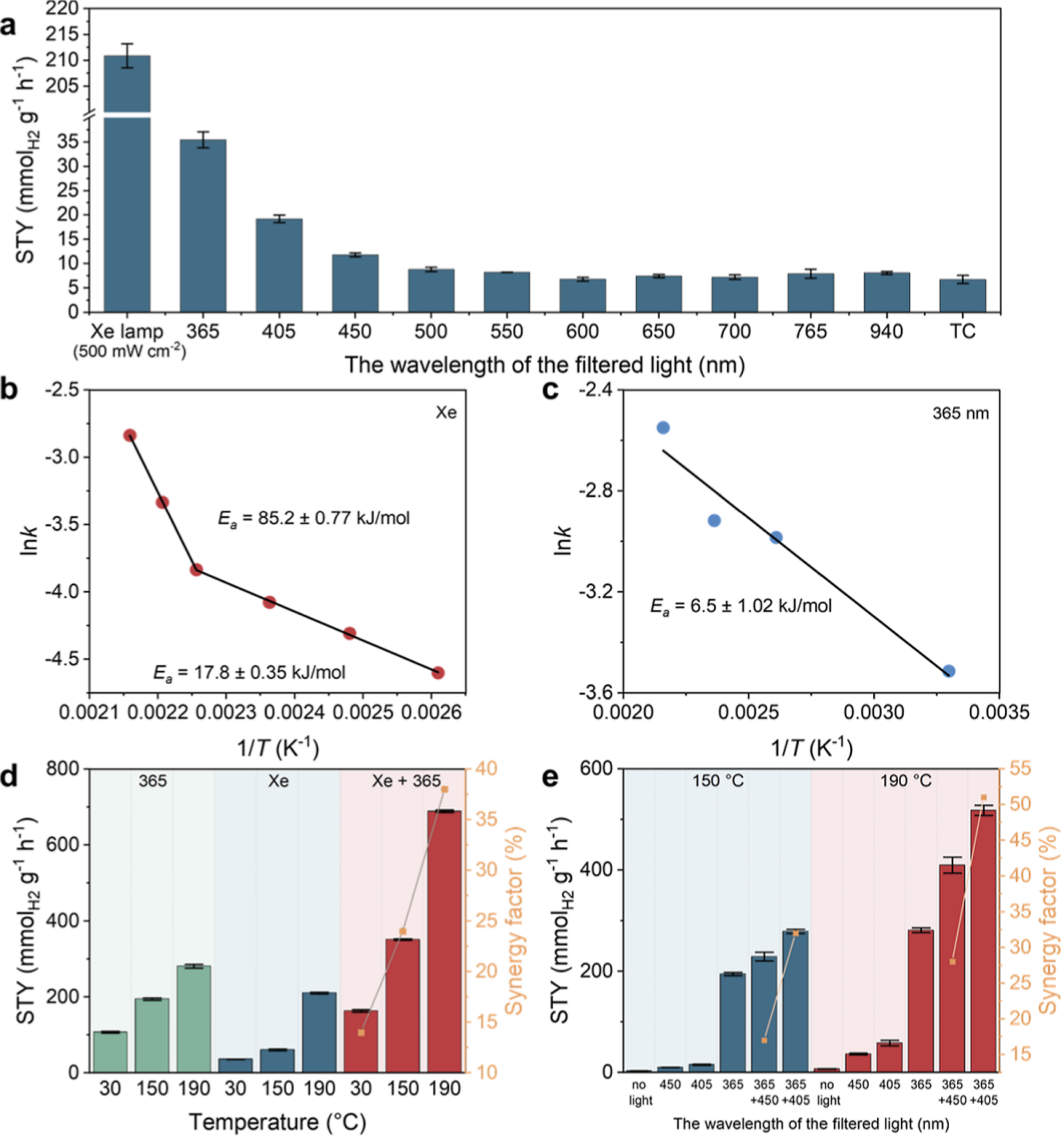
Photothermal catalytic performance and kinetic analysis of the temperature-regulated synergy. (a) Wavelength-dependent H_2_ space-time yield (STY), TC as thermal catalysis. (b, c) Arrhenius plots under (b) full-spectrum Xe lamp and (c) 365 nm illumination. (d) Temperature-dependent STY under 365 nm, Xe lamp, and combined illumination (Xe + 365 nm). (e) Synergistic effect of coilluminating 365 nm with 405 or 450 nm under constant photon flux (power densities adjusted to 221, 200, and 180 mW/cm^2^, respectively).

However, an intriguing phenomenon is observed in the visible region where irradiation at 405 and 450 nm sustains substantial H_2_ evolution rates of 19 and 11 mmol g^–1^ h^–1^, possessing photon energies of 3.06 and 2.75 eV that are theoretically insufficient to drive the band gap excitation of TiO_2_.^26^ In contrast, wavelengths exceeding 500 nm show negligible enhancement over the thermal baseline (thermal catalysis, TC). This distinct visible-light activity, decoupled from semiconductor band gap excitation, strongly implicates a secondary probable mechanism of hot-electron catalysis driven by hot-electron generation via interband transitions within the Pt nanoparticles.^11^ Consequently, the monochromatic contributions demonstrate that only UV and short-wavelength visible light (<500 nm), corresponding to semiconductor excitation and interband excitation of metal, can effectively participate in the synergistic catalysis. To decrypt the mechanistic underpinnings of this synergy, the reaction kinetics were systematically probed via Arrhenius analysis. Under monochromatic 365 nm illumination (pure semiconductor mode), the reaction follows a classical linear Arrhenius relationship across the entire temperature range, characterized by a constant, low apparent activation energy (*E*_a_) of 6.5 kJ mol^–1^. This linearity indicates that the UV-driven pathway operates via a consistent, barrier-limited mechanism dominated by photonic excitation.

In contrast, under Xe lamp illumination representing the combined semiconductor–metal excitation mode, the kinetics exhibit a striking nonlinear behavior, with a distinct inflection point emerging at approximately 170 °C. The Arrhenius plot splits into a low-temperature regime (<170 °C) with an *E*_a_ of 17.8 kJ mol^–1^, and a high-temperature regime (>170 °C) where the apparent *E*_a_ surges to 85.2 kJ mol^–1^ (Figures 2a,b, and S6). This convex Arrhenius behavior is compelling evidence that the dominant reaction pathway undergoes a fundamental transition at elevated temperatures under different light illumination. Moreover, the *E*_a_ regime shift signifies a catalytic process transition based on photon energy change. It suggests that thermal energy does not merely provide kinetic acceleration but actively triggers a high-efficiency, high-barrier catalytic cycle driven by hot-electron injection, distinct from the barrier-less UV pathway.^27–29^

To quantify the synergistic effect and exclude intensity-related artifacts, rigorous constant photon quantity tests were conducted (Figure 2d). Under these optimized conditions, this temperature-dependent nature was further quantified by analyzing the synergy factor (Figure 2d).^30^ At 30 °C, the synergistic enhancement is a modest 14% (Xe + 365 nm *vs* Xe and 365 nm). However, as the temperature increases to 190 °C, the combined illumination (Xe + 365 nm) yields an exceptional H_2_ production rate of 688 mmol g^–1^ h^–1^, which corresponds to a dramatic intensification of the synergy factor to 38% (compared to 24% at 150 °C) (Table S2). While the kinetic analysis confirms the existence of a thermal activation threshold for light of different wavelengths, it does not explicitly identify which specific optical channel is being regulated. A controlled dual-wavelength experiment was designed to isolate the interaction between the semiconductor channel (driven by 365 nm light) and the hot-electron injection channel (driven by visible light) under constant photon flux conditions (Figure 2e). Crucially, the varying response of different wavelengths to thermal stimuli acts as the definitive fingerprint of this mechanism. As the temperature is elevated from 150 to 190 °C, the system exhibits a starkly distinct thermal sensitivity depending on the excitation wavelength. Under strong excitation 405 nm coillumination with 365 nm, the synergy factor experiences a dramatic surge from 32% at 150 °C to 51% at 190 °C, corresponding to a robust thermal activation of the high-flux electron channel. Meanwhile, under weak excitation 450 nm coillumination with 365 nm, the system also displays a thermal response with the synergy factor showing an increase from 17% at 150 °C to 28% at 190 °C (Figure 2e and Table S3). This wavelength-dependent thermal amplification confirms that thermal energy acts as an indispensable thermodynamic factor permitting the injected hot electrons to effectively participate in the catalytic cycle.

### 3.3 Investigation of Electron Injection Mechanism

Direct spectroscopic observation of the generation and transfer dynamics of energetic hot electrons is required to validate this physical model. To elucidate the electronic nature of the catalyst, the electronic states were probed by high-resolution XPS (Figure 3a,b). Fresh Pt/TiO_2_ exhibits positive binding energy shifts (O 1s, 529.6 to 529.8 eV and Ti 2p, 458.4 to 458.6 eV, respectively) versus pristine TiO_2_, indicating Schottky-driven electron depletion.^31,32^ Crucially, the spent catalyst (Pt/TiO_2_–U) exhibits a reverse shift (O 1s, 529.8 to 529.5 eV and Ti 2p, 458.6 to 458.5 eV, respectively). Since structural reconstruction and sintering were rigorously ruled out via XRD and TEM (Figures S7 and S8), this negative binding energy shift signifies a substantial increase in surface electron density. This electronic evolution is attributed to the accumulation of photoinjected electrons that are trapped within the TiO_2_ lattice defects during the photothermal reaction.

**Figure 3:**
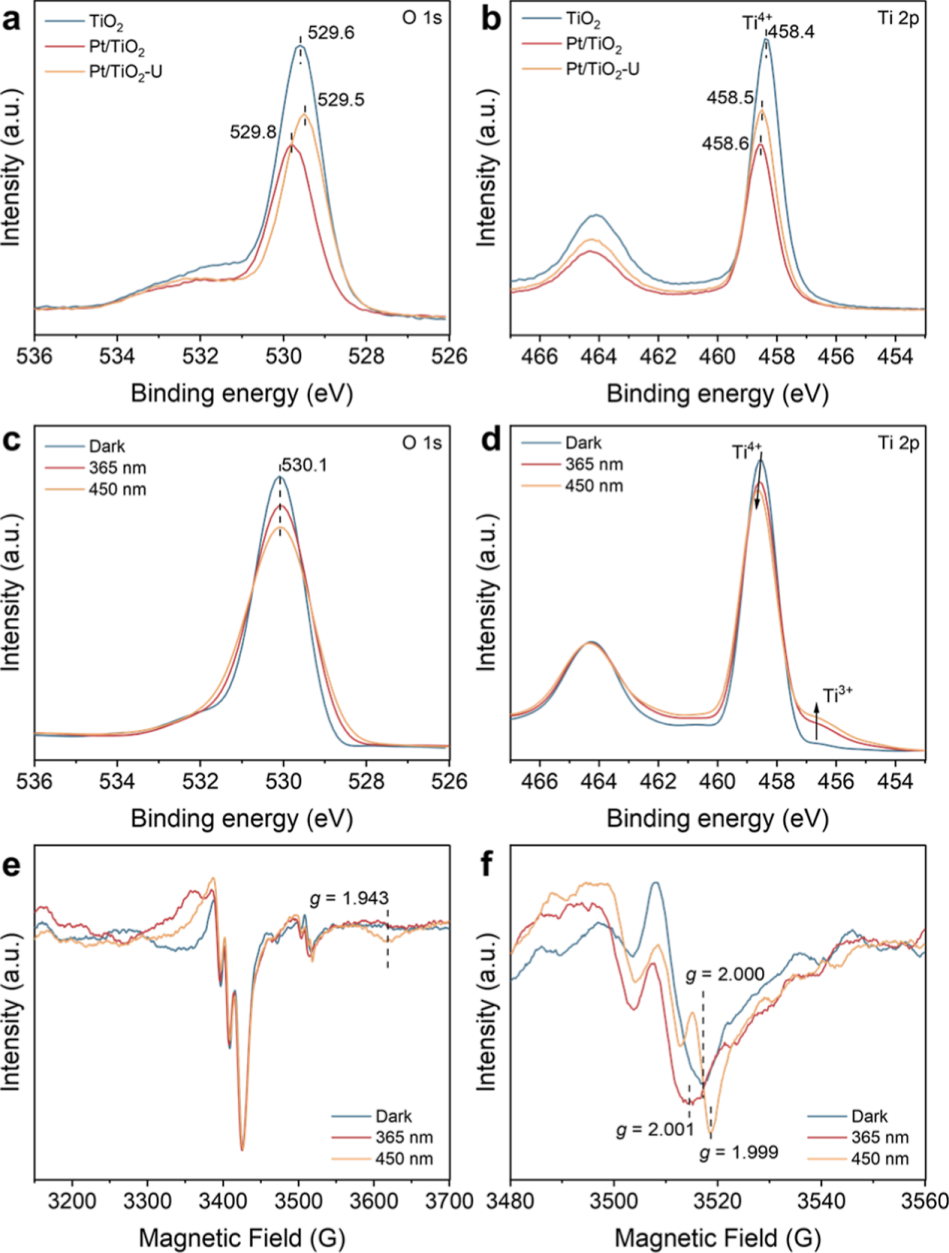
Spectroscopic evidence of charge carrier dynamics. (a,b) The XPS spectra of the TiO_2_, Pt/TiO_2_, and spent Pt/TiO_2_ (Pt/TiO_2_–U) of (a) O 1s and (b) Ti 2p. (c,d) In situ XPS spectra of (c) O 1s and (d) Ti 2p acquired under dark, 365 nm, and 450 nm illumination. (e) Full-range and (f) fine-scan in situ EPR spectra of the Pt/TiO_2_ under the same conditions.

In situ XPS shows the surface elements’ states under wavelength-resolved irradiation. The O 1s region (Figure 3c) confirms bulk lattice stability with modest intensification of the ∼531.5 eV peak at 365 nm but further broadening at 450 nm.^33^ Ti 2p spectra (Figure 3d) also exhibit wavelength-dependent behavior, where Ti^3+^ species (∼457.2 eV) emerge under 365 nm irradiation, while their population is enhanced at 450 nm.^22^ Furthermore, the in situ Pt 4f XPS spectra (Figure S9) and the ex situ fresh/spent spectra (Figure S10) exhibit nearly identical binding energies without obvious oxidative shifts. This stability indicates that during the continuous vectorial charge transfer, the rapid electron replenishment from methanol maintains the electronic balance of Pt, preventing its irreversible oxidation under rigorous photothermal conditions. Unlike UV excitation, where photogenerated carriers coexist and recombine in the lattice, hot-electron injection (450 nm) transfers electrons to TiO_2_ while confining holes on Pt, effectively suppressing recombination.^17^ In situ EPR under 365 nm irradiation shows the *g* = 2.001 signal assigned to trapped holes (O^–^ centers) from UV excitation (Figure 3e,f).^34^ In contrast, visible excitation (450 nm) produces a *g* = 1.999 signal characteristic of Ti^3+^ species,^35^ confirming hot-electron injection without concurrent hole signals. Furthermore, a *g* = 1.943 signal appears under visible light, attributed to bulk/subsurface electrons.^36^ This indicates hot electrons diffuse into the TiO_2_ bulk, distinct from UV-generated surface-confined carriers.

It is noteworthy that a superficial discrepancy exists between the in situ XPS and EPR results under 365 nm irradiation. While a Ti^3+^ shoulder is detected in XPS, the corresponding EPR signal is absent. The in situ XPS is conducted under ultrahigh vacuum (UHV) conditions, where simultaneous exposure to X-ray and UV irradiation inevitably induces surface oxygen desorption, thereby creating surface oxygen vacancies and permanent Ti^3+^ states.^37,38^ In contrast, in situ EPR captures the true steady-state charge-transfer dynamics. Under 365 nm illumination, photogenerated electrons in TiO_2_ transfer to the Pt electron sinks, depleting the localized trapped electrons in the TiO_2_ lattice and leading to an absent Ti^3+^ EPR signal. Conversely, the prominent Ti^3+^ EPR signal under 450 nm substantiates the reverse injection of hot electrons from Pt to TiO_2_.

These spectroscopic results confirm that visible excitation (450 nm) drives Ti^3+^ formation via hot-electron injection. The catalytic performance difference between strong excitation (405 nm) and weak excitation (450 nm) conditions results from the electron density, as the efficiency of hot-electron injection from Pt to TiO_2_ increases with photon energy.^39,40^ To validate this, the following kinetic studies directly compare the high-density 405 nm excitation against the low-density 450 nm excitation, isolating electron density as the critical variable for overcoming reaction barriers.

### 3.4 In Situ DRIFTS Study of Surface Intermediates

To visualize the molecular dynamics driven by these differing electron fluxes and identify the rate-determining steps, in situ DRIFTS was employed. Under 365 nm UV irradiation at 190 °C (Figure 4a), the spectrum displays a formate band located at ca. 1364 cm^–1^ and a persistent band accumulates at ca. 1750 cm^–1^, assigned to adsorbed formaldehyde (HCHO*).^41,42^ This suggests that even at 190 °C, UV irradiation cannot accelerate the rate-determining formaldehyde dissociation. The impact of UV irradiation and weak excitation was examined under 365 + 450 nm coillumination at 190 °C (Figure 4b). The formate band exhibits a shift from 1364 cm^–1^ to 1431 cm^–1^ compared with the UV-only condition, while the rate-limiting formaldehyde intermediate (1750 cm^–1^) almost vanished. The formate band of 365 + 450 nm coillumination matches the formate wavelength under visible-only conditions (Figure S11). This suggests that although weak excitation (450 nm) accelerates the conversion of formaldehyde, it traps the formate species in a metastable state,^43^ creating a new kinetic bottleneck that hinders the overall turnover rate. In contrast, when strong excitation is introduced (365 + 405 nm) at 190 °C (Figure 4c), the formaldehyde also vanishes, while formate species are maintained in their thermodynamically stable configuration (1364 cm^–1^).^44^ This implies that the high flux of hot electrons generated by 405 nm excitation effectively drives rapid catalytic turnover, successfully overcoming the barrier for formaldehyde.

**Figure 4:**
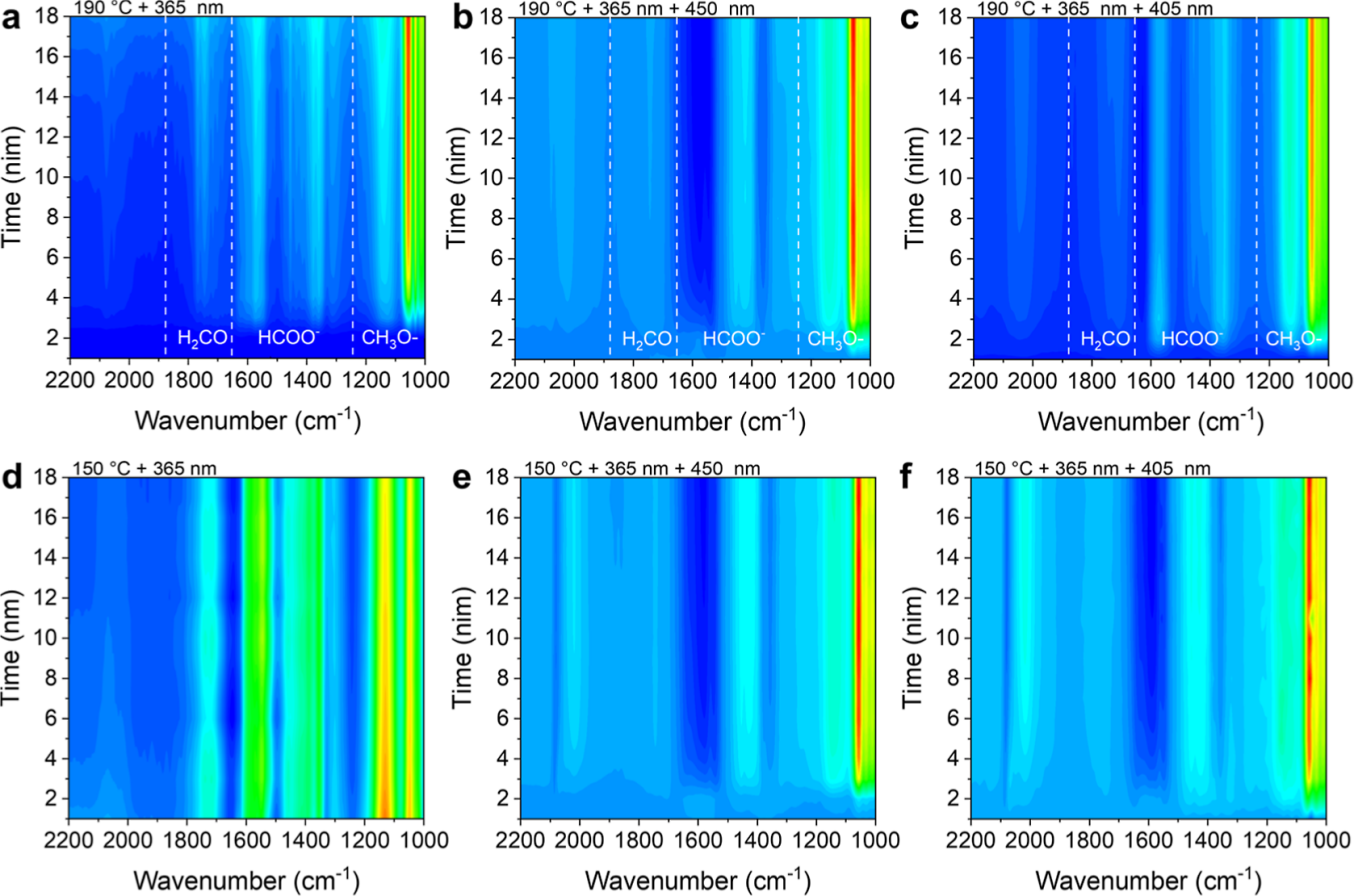
Molecular-level verification of the synergistic mechanism via in situ DRIFTS investigation. (a–c) The in situ DRIFTS of the Pt/TiO_2_ under (a) 365 nm, (b) 365 + 450 nm, and (c) 365 + 405 nm illumination at 190 °C. (d–f) The in situ DRIFTS of the Pt/TiO_2_ under (d) 365 nm, (e) 365 + 450 nm, and (f) 365 + 405 nm illumination at 150 °C.

To confirm the specific role of thermal energy in this synergy, control experiments were conducted at the lower temperature of 150 °C. The spectrum under UV-only illumination at 150 °C (Figure 4d) exhibits accumulation of formaldehyde (1750 cm^–1^) and the formate species at 1431 cm^–1^. Upon the introduction of visible light (both 365 + 450 nm in Figure 4e and 365 + 405 nm in Figure 4f), the formaldehyde band intensity decreases compared to UV alone. This experimental evidence proves that injected hot electrons are chemically active at 150 °C and can facilitate the dissociation of formaldehyde. However, the formate species remains trapped in the metastable state at 1431 cm^–1^. This reveals that while hot electrons drive the chemical bond breaking (HCHO dissociation), thermal energy is indispensable for the structural evolution (formate relaxation and desorption). Without sufficient heat (190 °C), the cycle remains bottlenecked at the formate conversion stage, preventing the high-efficiency turnover observed under photothermal synergy.

Under broadband Xe lamp irradiation at 190 °C (Figure S12), the spectrum mirrors the efficient 365 + 405 nm pathway, showing the stable 1364 cm^–1^ band and the absence of the 1750 cm^–1^ intermediate. This confirms that in the full solar spectrum, the strong visible-light excitation components effectively drive the reaction flux by overcoming the stabilization effect observed under weak excitation conditions.

### 3.5 Proposed Reaction Mechanism

Collective evidence reveals a temperature-regulated dual-channel synergistic mechanism. In the UV channel (Figure 5a), catalytic turnover is kinetically constrained by accumulation of formaldehyde intermediates (HCHO*), constituting the rate-determining step.^45^ Conversely, hot electron injection (Figure 5b) generates energetic hot electrons stored in the TiO_2_ lattice traps (bulk Ti^3+^), serving as an active site reservoir. However, at low temperatures, the catalytic efficiency remains limited. Without sufficient thermal energy, the reaction pathway is prone to being trapped in metastable configurations. The synergistic coupling of these pathways is strictly governed by thermal energy (Figure 5c). Heat provides the requisite activation energy to overcome the transport barrier for the trapped hot electrons.^46^ These energetic carriers populate surface active sites (likely Ti^3+^), facilitating activation and conversion of intermediates. This thermally facilitated evolution circumvents kinetic bottlenecks, integrating efficient photon harvesting with TiO_2_ surface chemistry for supra-additive performance. The fate of the photogenerated holes (*h* ^+^) left in the TiO2 valence band is scavenged by the methanol molecules. This anodic half-reaction triggers the initial dehydrogenation step, oxidizing methanol into surface methoxy intermediates (CH_3_O*) and releasing protons (*h* ^+^). This hole-scavenging process supplies the primary methoxy precursors, which are consistent with our in situ DRIFTS observations.

**Figure 5:**
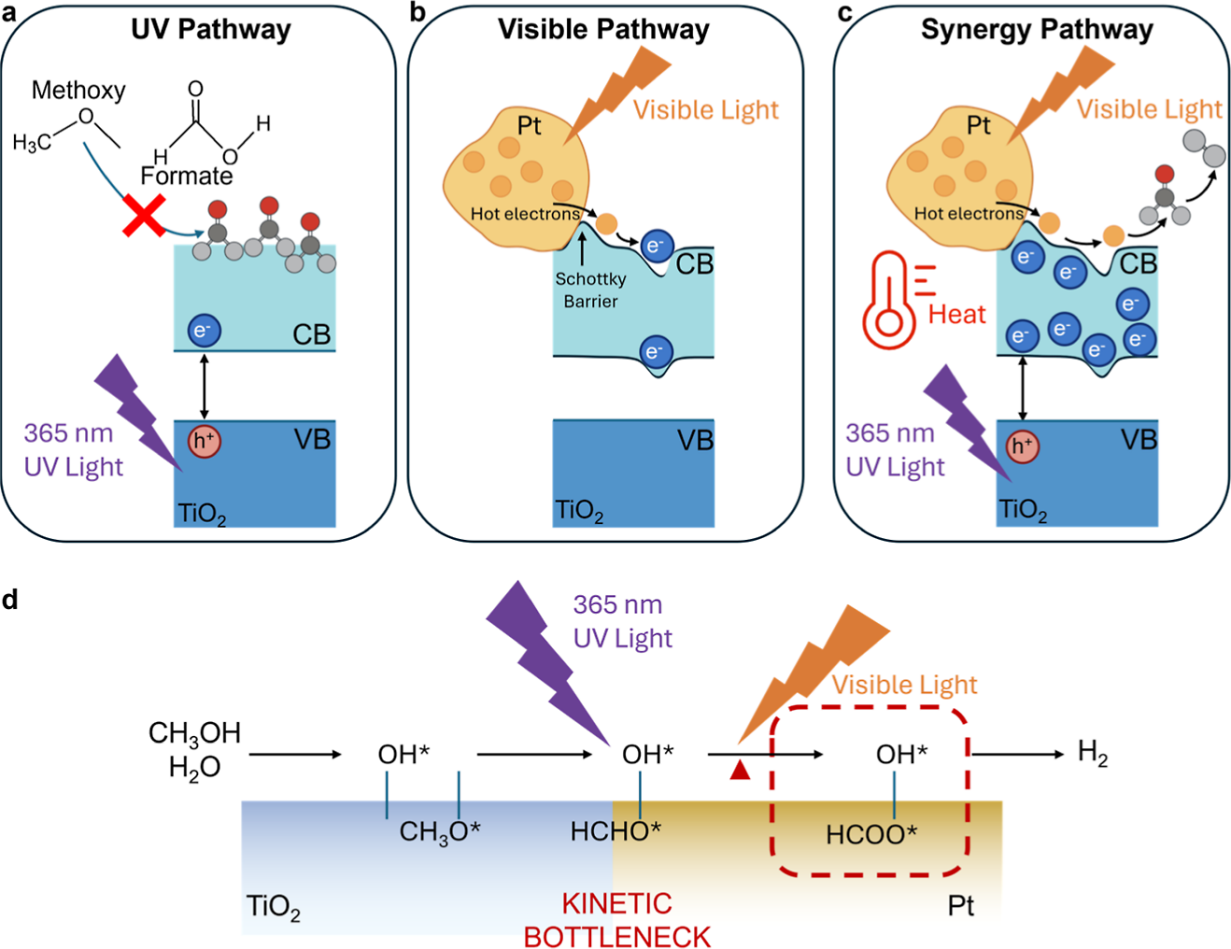
Proposed temperature-regulated photothermal mechanism. (a–c) Schematics of the UV-semiconductor, hot electron injection channel, and synergistic pathways. (d) Molecular reaction scheme. The UV pathway creates a kinetic restriction of accumulated formaldehyde (red box). Thermal energy (red triangle) regulates the injection of energetic hot electrons to clear this restriction, driving the rate-determining C–H bond cleavage.

Based on the analysis above, a comprehensive mechanism illustrating the temperature-regulated dual-channel synergy is proposed in Figure 5d. Under UV irradiation, the semiconductor pathway on TiO_2_ utilizes valence band holes (*h* ^+^) to drive the oxidation of methoxy (CH_3_O*) to formaldehyde (HCHO*).^33,47^ However, further dissociation of this intermediate is kinetically hindered by insufficient activation energy, causing surface accumulation and kinetic restriction.^48^ Upon visible light irradiation and thermal energy, the hot-electron injection pathway on Pt is engaged. Hot electrons (e_hot_^–^) generated on Pt facilitate the cleavage of the rate-limiting C–H bond in accumulated HCHO*, converting it to formate (HCOO*). Eliminating this critical rate-determining step accelerates the catalytic cycle, enabling the exceptional hydrogen production.

The UV-excited TiO_2_ holes drive the initial methanol to formaldehyde oxidation—a thermodynamically demanding step that injected hot electrons cannot initiate alone. While this sustains catalytic turnover, it is bottlenecked by formaldehyde accumulation. In contrast, the visible pathway overcomes the formaldehyde dissociation barrier but lacks photogenerated holes to supply this intermediate. Therefore, UV generates reactive precursors while visible light facilitates the rate-determining step, though this strictly requires high-flux 405 nm (not weak 450 nm) excitation to effectively couple hot electrons with thermal activation. This functional complementarity yields supra-additive performance.

## 4 Conclusion

In summary, this work establishes a temperature-regulated dual-channel framework, achieving a 688 mmol g^–1^ h^–1^ hydrogen evolution rate over Pt/TiO_2_. Kinetic analysis reveals nonlinear Arrhenius behavior, identifying thermal energy as a critical thermodynamic regulator of the transition from photon-limited semiconductor mode to hot-electron injection mode. These findings reveal a hierarchy of thermal responsiveness. The UV pathway (365 nm) generates intermediates but is kinetically limited, showing only structural thermal response. Strong excitation (405 nm) exhibits a profound kinetic thermal response, enabling activation of high-flux hot electrons to eliminate reaction bottlenecks (>170 °C). The weak excitation pathway (450 nm) partially remains kinetically restricted as its limited electron flux to sufficiently couple with the thermal activation. Ultimately, this study moves beyond simple photothermal heating, establishing that the catalytic performance arises from the coupling of semiconductor band gap excitation and metal interband excitation under different temperature. These findings offer a transformative blueprint for designing next-generation catalysts capable of rationally converting broadband solar energy into targeted chemical bonds.

## Supplementary Material



## Notes

HAADF-STEM and TEM images of catalysts; H_2_-TPR and H_2_-TPD spectra of catalysts; the surface temperature of catalysts; wavelength-dependent and temperature-dependent H_2_ STY of catalyst; XRD of catalysts; in situ XPS spectra of catalysts; and in situ DROFTS of catalyst (PDF)
