# Supplementary material for: Spectrally-Resolved Synergy in Photothermal Catalysis: A Temperature-regulated Transition of Hot-electron Transfer for Methanol Steam Reforming
Source: ACS Omega. 2026 Jun 18;11(25):38242–51. doi: 10.1021/acsomega.6c05080 (PMC13325390; doi:10.1021/acsomega.6c05080)
Supplement: Supplementary file 1 [file ao6c05080_si_001.pdf]

Supporting Information

Spectrally-Resolved Synergy in Photothermal Catalysis: A Temperature-Regulated Transition of Hot-Electron Transfer for Methanol Steam Reforming

Lifeng Xu,[a] Chenghao Yao,[b] Rui Lang,[b] Lei Li *[b] Zhan Lin*[b]

[a] *College of Chemical and Biological Engineering, Zhejiang University, Hangzhou, 310058, China*

[b] *School of Chemical Engineering and Light Industry, Guangdong University of Technology, Guangzhou, 510006 China*

E-mail: li.lei@gdut.edu.cn, zhanlin@gdut.edu.cn

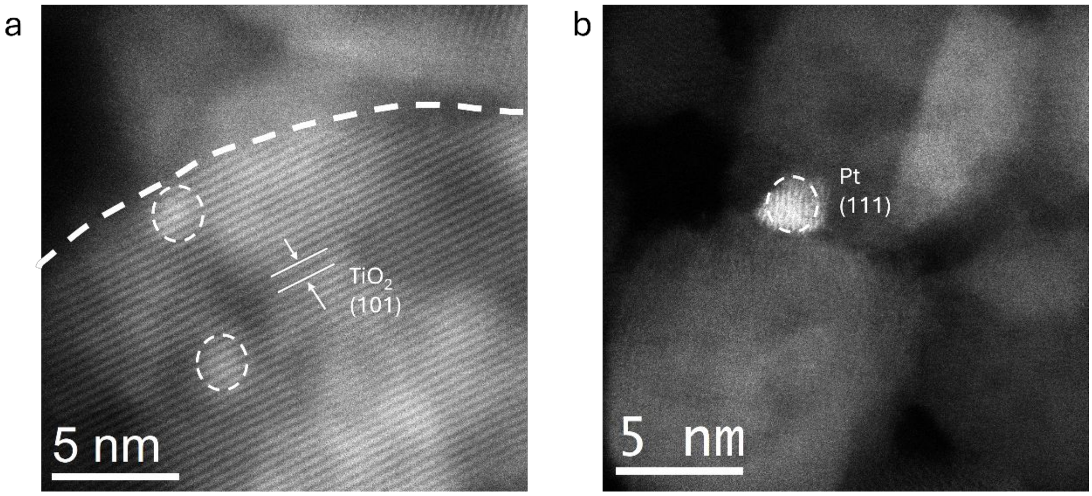

Figure S1. HAADF-STEM images of Pt/TiO_2 (a) before and (b) after reaction.

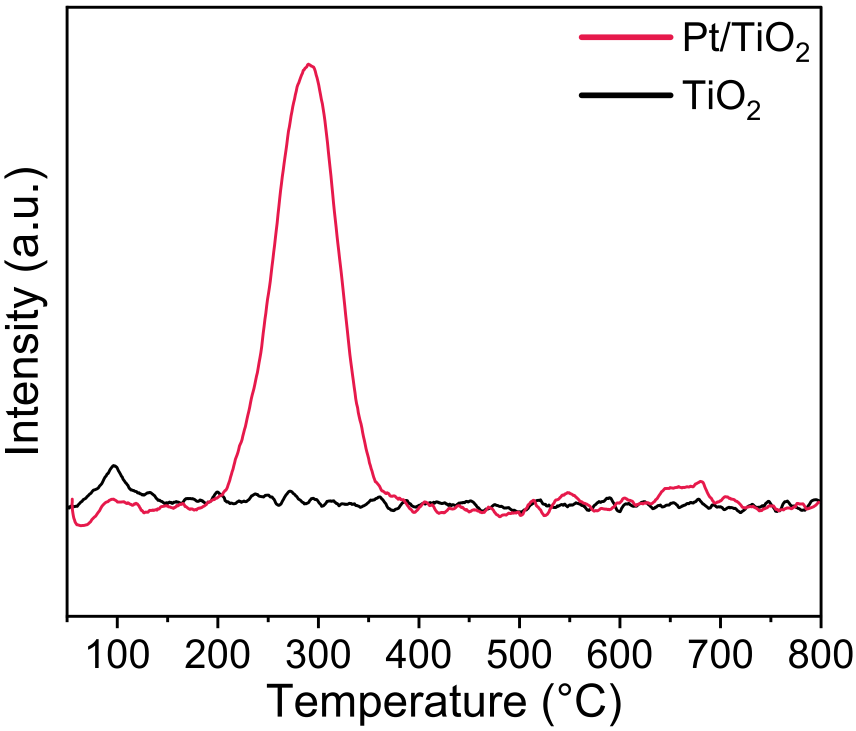

Figure S2. H_2-TPR spectra of the catalysts

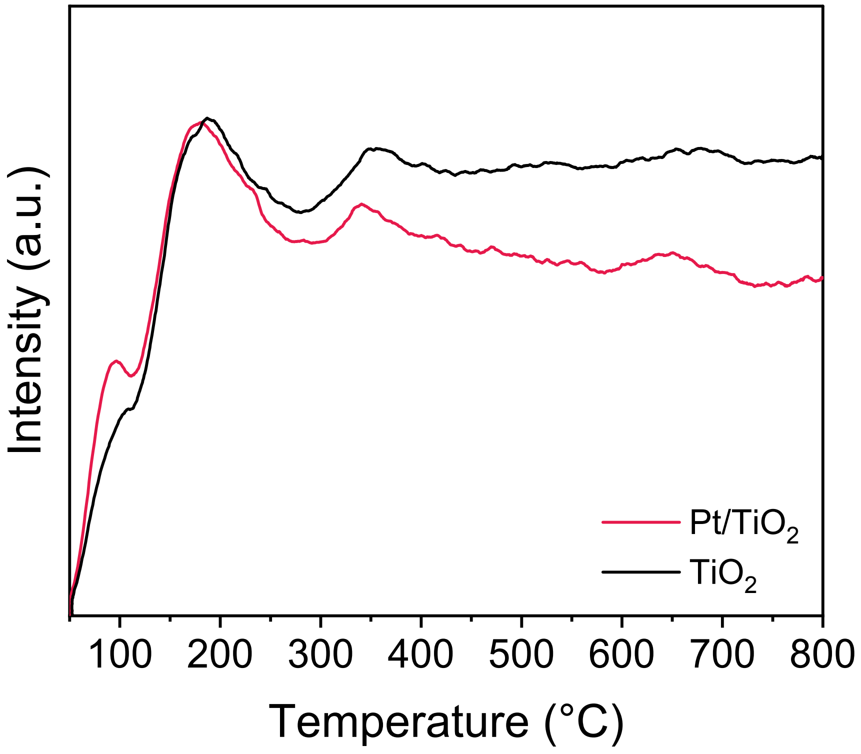

Figure S3. H_2-TPD spectra of the catalysts

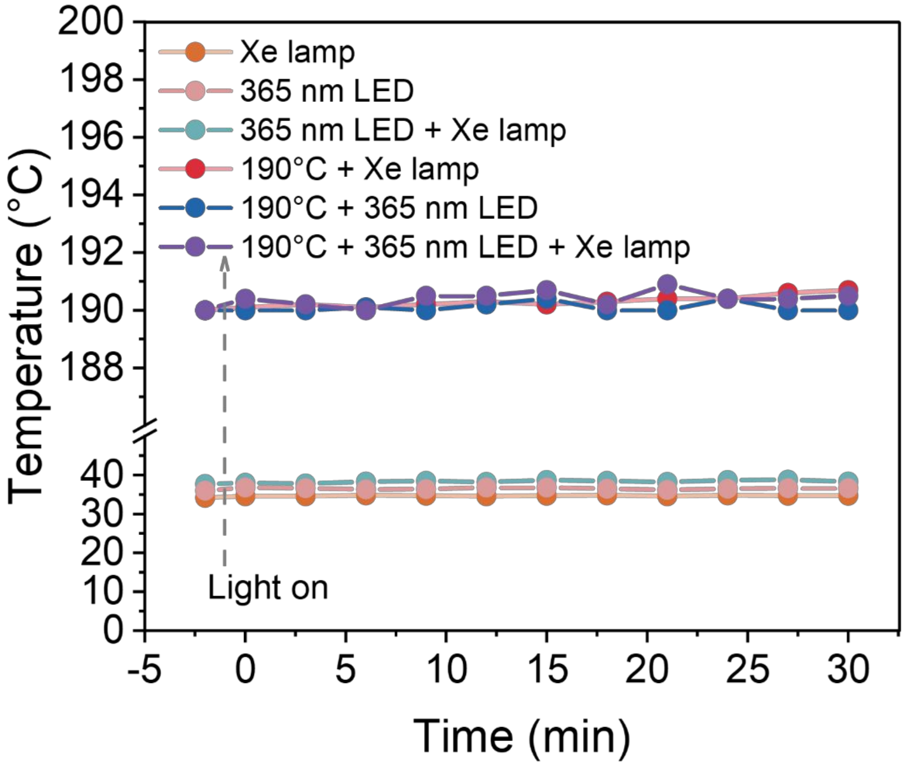

Figure S4. The surface temperature mapped by an infrared camera of the catalyst under fixed temperature

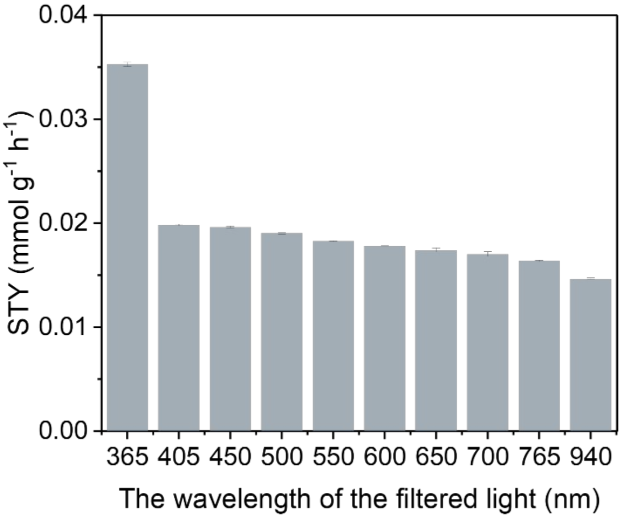

Figure S5. Wavelength-dependent H_2 space-time yield (STY) of TiO_2.

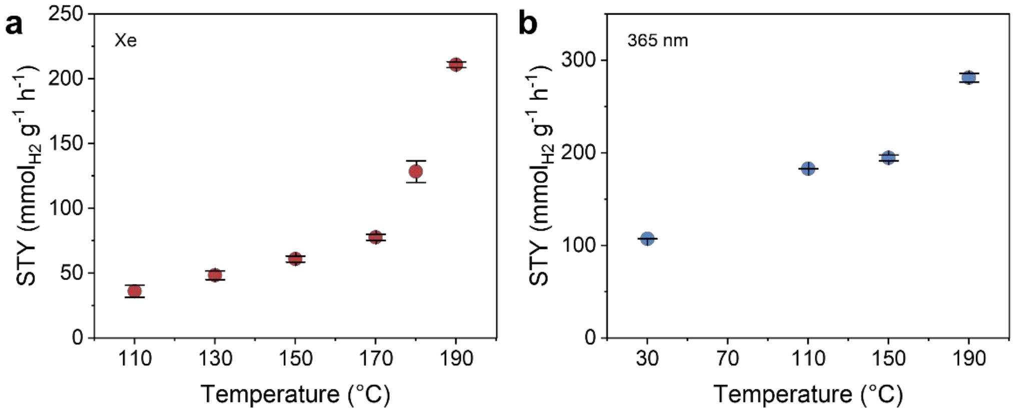

Figure S6. Temperature-dependent STY under 365 nm and Xe lamp illumination.

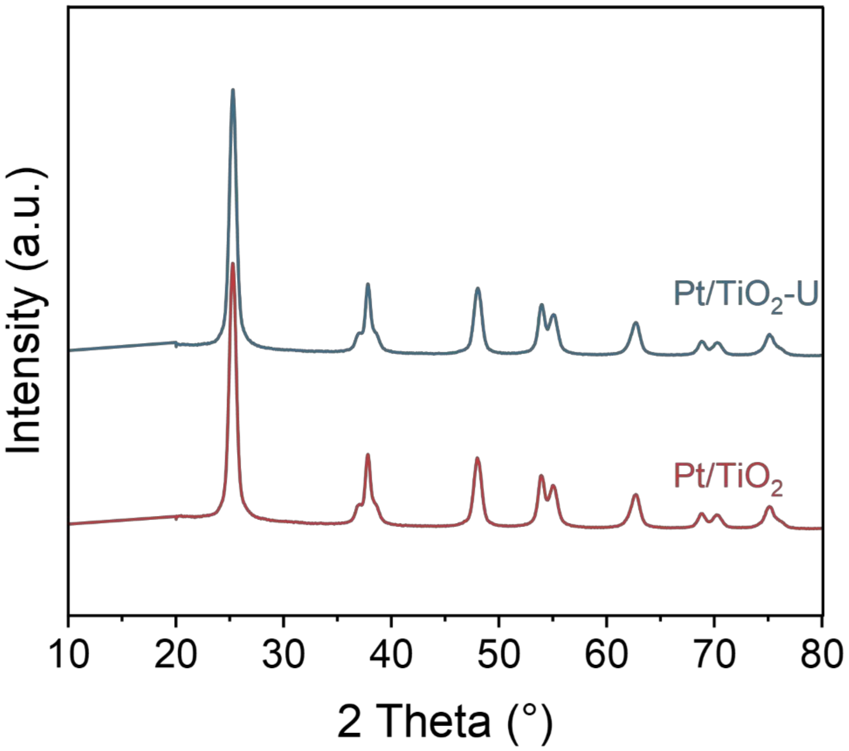

Figure S7. XRD patterns of the fresh and spent catalysts.

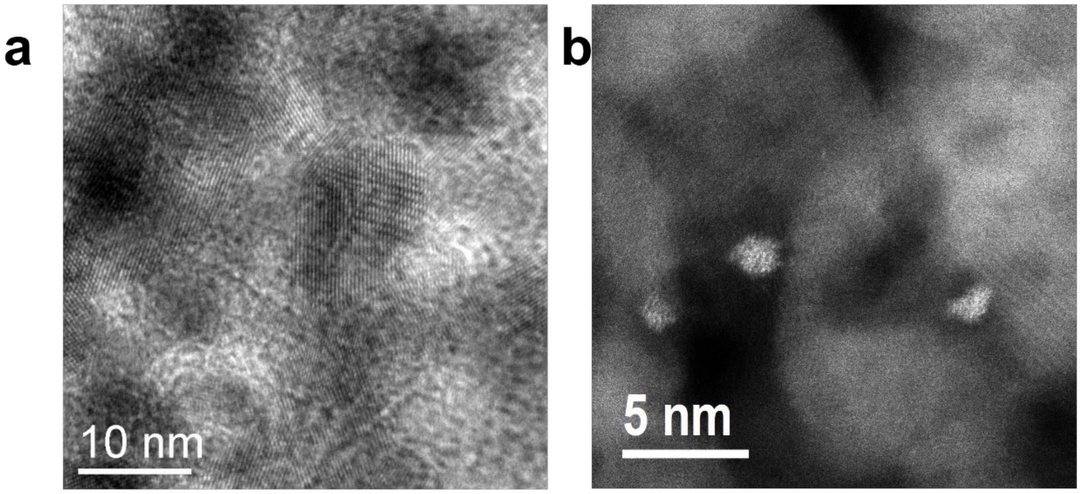

Figure S8. **(a)** TEM and **(b)** HAADF-STEM images of the Pt/TiO_2.

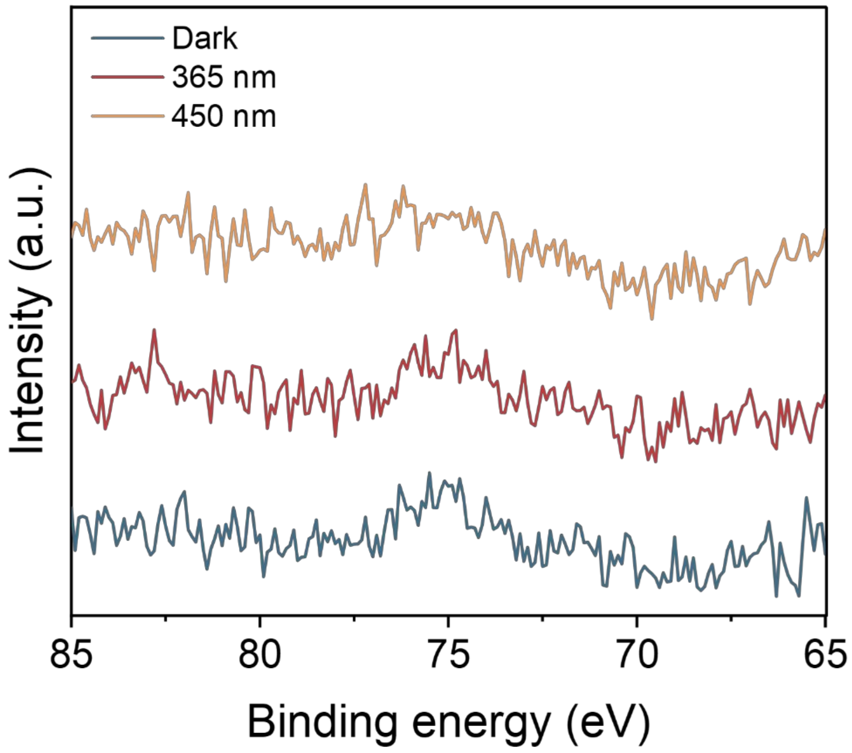

Figure S9. In situ XPS spectra of Pt 4f acquired under dark, 365 nm, and 450 nm irradiation.

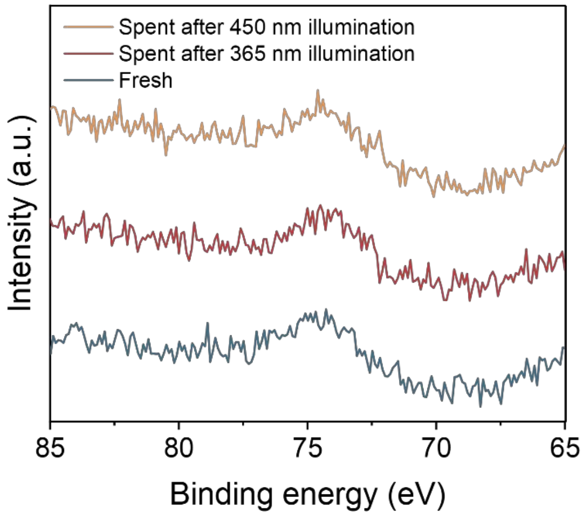

Figure S10. High-resolution Pt 4f XPS spectra of the fresh and spent Pt/TiO_2 catalysts.

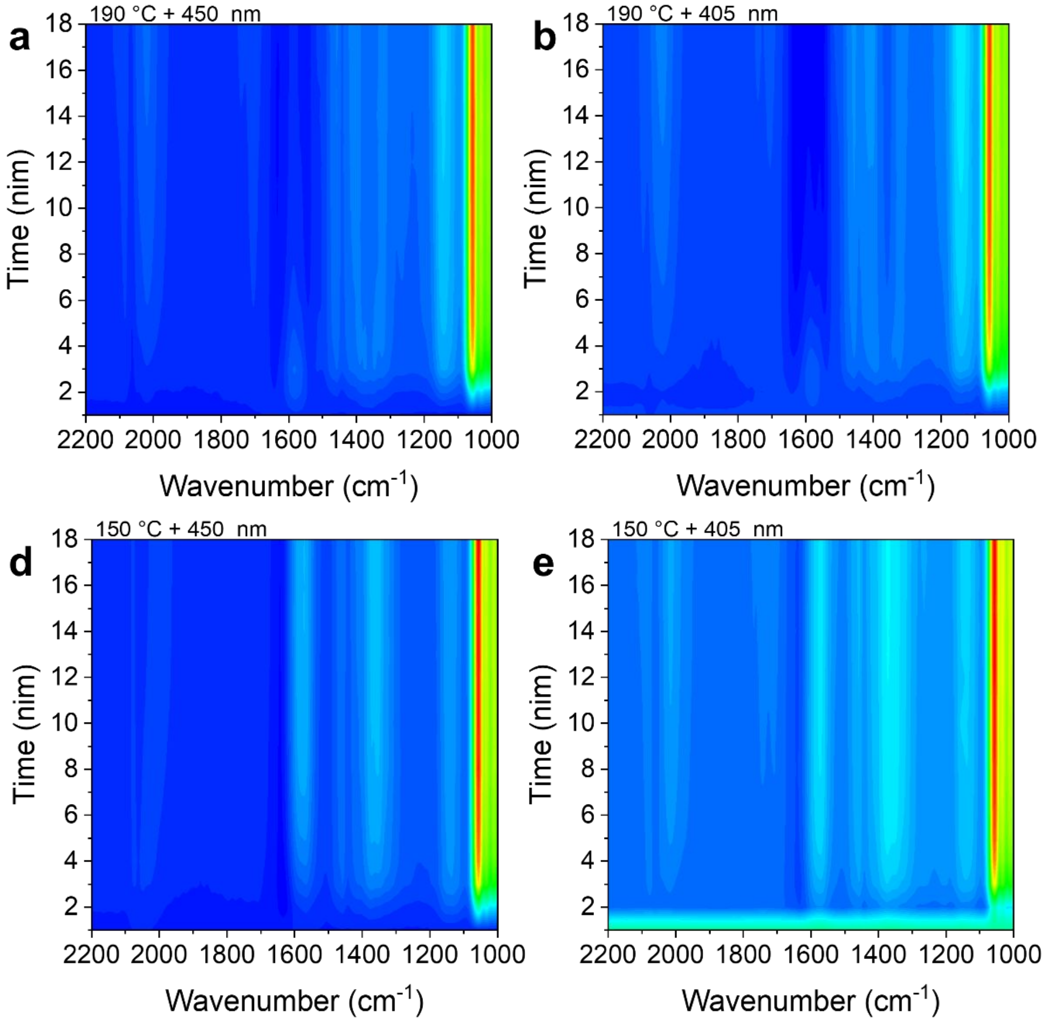

Figure S11. The *in situ* DRIFTS of Pt/TiO_2 under 405 nm and 450 nm illumination at 150 °C and 190 °C.

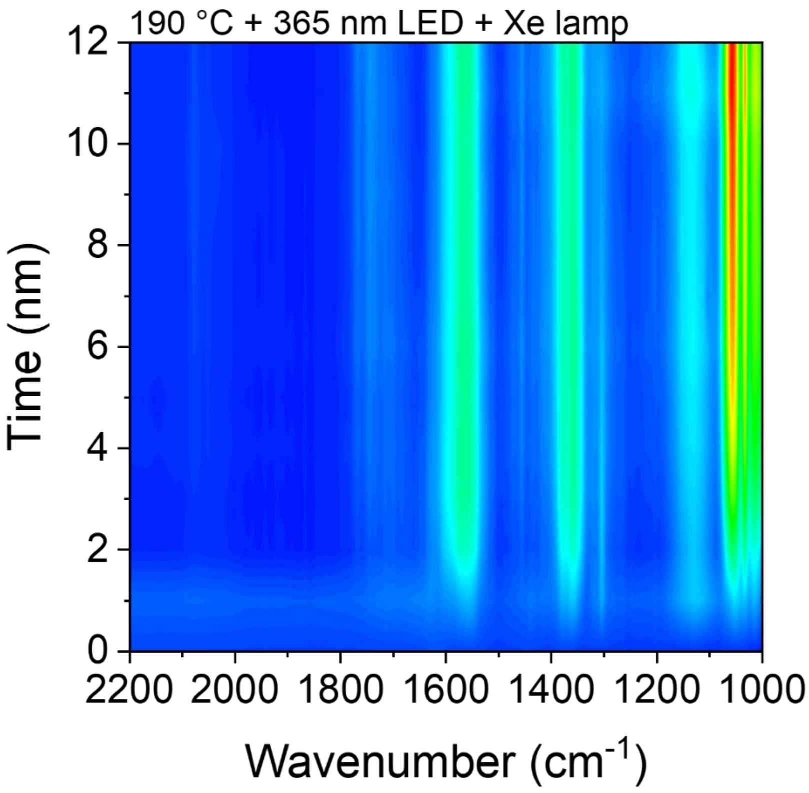

Figure S12. The *in situ* DRIFTS of Pt/TiO_2 under 365 nm and Xe illumination at 190 °C.

Table S1. The physicochemical characteristics of the catalyst.

	m_0 (g)	V_0 (mL)	Elemental	C_o (mg/L)	f	C_1 (mg/L)	C_x (mg/kg)	W
1	0.0526	10	Pt	2.8671	1	2.8671	545.08	0.0545%

Table S2. The Synergy Factor of Xe and 365 nm irradiation.

	Temperature (°C)	Synergy Factor (%)
1	190	38
2	150	24
3	30	14

Table S3. The Synergy Factor of 365, 405, and 450 nm irradiation.

	Temperature (°C)		Synergy Factor (%)
1	190	365+450	28
2		365+405	51
3	150	365+450	17
4		365+405	32
